# Dimeric dipeptide mimetics of the nerve growth factor Loop 4 and Loop 1 activate TRKA with different patterns of intracellular signal transduction

**DOI:** 10.1186/s12929-015-0198-z

**Published:** 2015-12-08

**Authors:** Tatyana A. Gudasheva, Polina Yu Povarnina, Tatyana A. Antipova, Yulia N. Firsova, Mark A. Konstantinopolsky, Sergey B. Seredenin

**Affiliations:** Department of Medicinal Chemistry, V.V. Zakusov Institute of Pharmacology, Baltiyskaya str. 8, 125315 Moscow, Russia; Laboratory of Neuroprotective Pharmacology, V.V. Zakusov Institute of Pharmacology, Baltiyskaya str. 8, 125315 Moscow, Russia; Laboratory of Pharmacological Regulation of Alcohol and Drug Addiction, V.V. Zakusov Institute of Pharmacology, Baltiyskaya str. 8, 125315 Moscow, Russia; Department of Pharmacogenetics, V.V. Zakusov Institute of Pharmacology, Baltiyskaya str. 8, 125315 Moscow, Russia

**Keywords:** Nerve growth factor, GK-2, GK-6, PI3K/AKT, MAPK/ERK

## Abstract

**Background:**

This study aimed at developing nerve growth factor (NGF) mimetics that selectively activate specific biological signals and, as a result, lack the side effects of the full-length protein. Two dimeric dipeptides, bis-(N-aminocaproyl-glycyl-L-lysine) hexamethylenediamide (GK-6) and bis(N-succinyl-L-glutamyl-L-lysine) hexamethylenediamide (GK-2), were designed based on the most exposed outside fragments of NGF, namely, the loop 1 and loop 4 β-turn sequences, respectively. These dipeptides exhibited neuroprotective activity in vitro at micro-nanomolar concentrations.

**Results:**

Studies on the mechanism of action revealed that both compounds elevate the level of tyrosine kinase A (TrkA) receptor phosphorylation and that they each have different postreceptor signaling patterns. GK-6 increases the levels of extracellular signal-regulated kinase (ERK) and AKT kinase phosphorylation, whereas GK-2 only increases the level of AKT phosphorylation. Apart from the neuroprotective activity, GK-6 promoted differentiation in PC12 cells, whereas GK-2 did not. Furthermore, it was established that the neuroprotective activity of GK-2 was completely abolished by a selective inhibitor of phosphatidylinositol 3-kinase (LY294002) but not by a specific inhibitor of mitogen-activated protein kinases MEK1 and MEK2 (PD98059). In vivo experiments demonstrated that GK-2 did not induce hyperalgesia, which is one of the primary adverse effects of NGF. By contrast, GK-6 produced a significant decrease in the pain threshold of rats as determined by the tail flick test.

**Conclusion:**

The data obtained suggest that dimeric dipeptide NGF mimetics are promising candidates in the development of pharmacological agents with NGF-like activity that are free of the main side effect of NGF.

## Background

Nerve growth factor (NGF), a member of the neurotrophin family, is essential for the development and survival of several populations of neurons and a number of nonneural cells. Despite this factor’s considerable therapeutic potential, the clinical application of NGF is limited by its strong side effects, the most important of which are hyperalgesia and weight loss [1].

NGF exerts its main effects by interaction with the TrkA transmembrane receptor. Activation of TrkA by NGF triggers signal transduction cascades involving phosphatidylinositol 3-kinase/AKT (PI3K/AKT) and mitogen-activated protein kinase/extracellular-signal-regulated kinases (MAPK/ERK) pathways. The PI3K/AKT pathway is involved in the regulation of cell survival but not in the differentiation and formation of neurites [2]. The MAP-kinase pathway is associated with neuroprotection and differentiation and appears to be involved in hyperalgesia [3]. The design of small, proteolytically stable NGF mimetics that exert defined biological activities via the selective activation of TrkA-mediated signaling might provide a useful approach for the development of therapeutic agents for several diseases applications [2, 4].

We formed the working hypothesis [5] that by interacting with the same receptor, multiple neurotrophin hairpin loops can activate various intracellular signaling cascades and are therefore responsible for an array of neurotrophin effects. Within the framework of this hypothesis, the dimeric dipeptide bis(N-succinyl-L-glutamyl-L-lysine) hexamethylenediamide (GK-2) was designed based on the NGF loop 4 β-turn sequence Asp93–Glu94–Lys95–Gln96, which is the most exposed fragment and therefore may play a major role in the interaction of NGF with the receptor. We included the central fragment of the β-turn, Glu94–Lys95, in the dipeptide composition. The residue Asp93 was substituted by its bioisostere, a succinic acid residue, and Gln96 was substituted by an amide group. The purpose of these two substitutions was to stabilize the β-turn conformation and to increase the resistance of the compound to peptidases. Because NGF interacts with the TrkA in the homodimer form, we linked two β-turn mimetics by a hexamethylene diamine spacer. The dimeric dipeptide bis-(N-aminocaproyl-glycyl-L-lysine) hexamethylenediamide (GK-6) was designed analogously to GK-2 based on the NGF loop 1 β-turn (RU Patent №2410392, 2010; US Patent Appliction №US 2011/0312895 A1).

It has been shown in vitro, using both immortalized and primary cell cultures, that GK-2 and GK-6 exert NGF-like neuroprotective activity (10uM-1nM) [5, 6]. Maximal neuroprotective effects were observed at concentrations of 1uМ (GK-6) and 10nМ (GK-2); therefore, these concentrations were used for further in vitro experiments.

The neuroprotective activity of GK-2 at doses of 0.1-1 mg/kg (i.p.) was also determined in animal models of cerebral ischemia [7, 8] and in a model of rat traumatic brain injury [9].

Herein, we report a comparative study of the NGF loop 1 and NGF loop 4 β-turn mimetics, GK-6 and GK-2, respectively. We established that both peptides activate TrkA receptors but showed different patterns of intracellular signal transduction.

## Methods

### Drugs and reagents

The dimeric dipeptides GK-6 and GK-2 were synthesized on the base of murine NGF at the Zakusov Institute of Pharmacology (Moscow, Russia).

Inhibitors of PI3K (LY294002) and MAPK (PD98059) were purchased from Tocris Bioscience (Bristol, UK). The tetrazolium dye 3-(4,5-dimethylthiazol-2-yl)-2, 5-diphenyltetrazolium bromide (MTT),was obtained from Sigma-Aldrich (St. Louis, MO, USA). Dulbecco’s Modified Eagle’s medium was purchased from HyClone Laboratories (Logan, UT, USA). Fetal bovine serum was obtained from Gibco (Langley, OK, USA). Glutamine was purchased from ICN Pharmaceuticals. Inc. (Costa Mesa, CA, USA). Poly-D-lysine was purchased from BD Biosciences (San Jose, CA, USA). DC protein assay was purchased from Biorad (Hercules, CA, USA). Anti-TrkA, anti-pTrkA, anti-AKT1/2/3, anti-pAKT1/2/3, anti-ERK1/2, anti-pERK1/2 antibodies and enhanced chemiluminescence kits were obtained from Santa Cruz Biotechnology (Dallas, TX, USA). Anti-β-actin antibodies and horseradish peroxidase conjugated antibodies were purchased from Abcam (Cambridge, MA, USA).

### Cell cultures

Cells were maintained at 37 °C in Dulbecco’s Modified Eagle’s medium, 10 % fetal bovine serum, 2 mM glutamine, 5 % CO_2_ and 95 % air at 37 °C and passaged by trypsinization. Rat hippocampal neurons were taken from 18-day-old fetuses using techniques previously described [10]. Cells were placed on 48-well plastic plates previously treated with poly-D-lysine (5 mkg/1 cm2) at a plating density of 350x103 cells per well. The cultures were maintained in a humidified atmosphere of 5 % CO_2_ and 95 % air at 37 °C.

### Western blot analysis

The mouse hippocampal HT-22 cells were plated into 6-well plates at 200 x 103 cells per well. Fifteen, 30, 60 and 180 min after incubation with GK-6 (10^−6^M), GK-2 (10^−8^M) or NGF (10^−9^M) [11], cells were collected, and protein was extracted for Western blot analysis. Samples were homogenized in a lysis buffer (50 mM Tris–HCl, pH = 7.5, 5 mM EDTA, 1 mM DTT, 1 % Triton X-100 supplemented with protease and phosphatase inhibitor cocktail), incubated on ice for 5 min and then centrifuged (13 000 rpm, 10 min, at 4 °C). Protein levels of the supernatant lysates were measured using the DC protein assay. Proteins were separated in a 10 % SDS-PAGE gel and blotted for 1 h (15 V) onto a PVDF membrane. Membranes were incubated at 4 °C overnight with the following primary antibodies: anti-pTrkA, anti-pAKT1/2/3, anti-pERK1/2, anti-TrkA, anti-ERK1/2, and anti-AKT1/2/3. All of the antibodies were used at 1:1000 final dilutions in 0.5 % non-fat dry milk in TBST. Equal loading was confirmed using anti-.-actin (1:5000 in 0.5 % non-fat dry milk in TBST). Membranes were washed with TBS/0.5 % Tween (TBST) and incubated with horseradish peroxidase conjugated secondary antibody (1:20000 in 0.5 % non-fat dry milk in TBST). Secondary antibodies tagged to peroxidase were used to visualize immunoreactive bands using enhanced chemiluminescence kits.

### Measurement of PC12 cell morphological differentiation (neurite outgrowth)

NGF mimetics were tested for their ability to induce neurite outgrowth in PC12 cells. The cells were treated with GK-6 (10^−6^M), GK-2 (10^−5^ and 10^−8^M) or NGF (10^−9^M) 3 times per 48-h time period. An Eclipse TS100-F light microscope (Nikon, Tokyo, Japan) equipped with a phase- contrast condenser, a 20X objective lens and a digital camera (Canon, Tokyo, Japan) was used to capture images using the manual setting. To analyze the number and length of neurites, approximately 100 cells were counted from at least 10 randomly chosen visual fields for each culture. Using the Photoshop software program (Adobe, San Jose, CA, USA), the number and length of neurites were analyzed. The cells were scored as differentiated if one or more neurites were longer than the diameter of the cell body.

### Cell viability assay

To examine whether GK-2 exerts neuroprotective activity through AKT signaling, we used LY294002, a specific inhibitor of PI3K, and the MAPK inhibitor PD98059. Concentrations of 100 μM and 50 μM were selected for LY294002 and PD98059, respectively [12, 13]. The hippocampal cells HT-22 were pre-incubated with LY294002 and PD98059 for 30 min before treatment with GK-2 (10^−5^M and 10^−8^M) or NGF (10^−9^M) for 24 h. The cells were then exposed to oxidative stress (1.5 mM H2O2 for 30 min) [14], and cell survival after 30 min was measured at 37 °C using the MTT assay [15].

### Animals

Both male Wistar rats (300–380 g) and outbred rats (250–300 g) were purchased from the Animal Breeding Facility at the Institute of Bioorganic Chemistry in Pushchino (Moscow, Russia). The animals were housed under natural light–dark cycling conditions with food and water provided ad libitum. All experimental procedures were performed in accordance with the requirements of the Directive 2010/63EU of the European Parliament and the Council of 22 September 2010 on the protection of animals used for scientific purposes and were approved by the Institutional Animal Care and Use Committee of Zakusov Institute of Pharmacology (Meeting Protocol №1 from 10.11.2014).

### Tail flick test

In two separate experiments with GK-6 and GK-2, 36 and 40 male outbred rats were used, respectively. In these groups, each control or experimental subgroup was composed of 9–10 animals. The experiments were performed from 5 to 8 p.m. The effects of peptides on the pain thresholds were assessed via the immersion “tail flick” test in rats [16]. The animals were habituated to individual Plexiglas cylindrical restrainers for 20–30 min. Next, thermal noxious stimuli were applied by immersing the distal third of each rat's tail in hot water (55 ± 0.2 °C), and the latencies of reflex were measured. Dipeptides GK-6 and GK-2 dissolved in distilled water were administered i.p. at doses of 0.5, 1.0 and 2.0 mg/kg. Control animals were injected with an equal volume of vehicle. Tail flick latency was measured 3–4 times for 30 min in 10-min intervals before GK-6 or GK-2 administration (the mean baseline level) and 30, 60 min and 24 h after dipeptide administration. The time intervals were selected based on data related to NGF-induced hyperalgesia [17]. The cutoff time for latencies was set to 20 s to avoid skin damage.

### The study of possible effects of GK-2 and GK-6 on the body weight of rats

Male Wistar rats were used. Separate experiments were performed with GK-6 and GK-2. In each experiment, the rats were randomly assigned to the following two groups: the control group (*n* = 10) and the GK-6 group (*n* = 10) in the first experiment and the control group (*n* = 12) and the GK-2 group (*n* = 13) in the second experiment. GK-6 and GK-2 (dissolved in distilled water) were administered daily at doses of 2 mg/kg and 0.5 mg/kg i.p., respectively, for 2 weeks. These doses were chosen as the most pharmacologically active doses based on preliminary studies. Control animals received i.p. injections of distilled water. The body weight of the rats was measured every 3–4 days during the experiment.

### Statistical methods and data analysis

Kruskal-Wallis ANOVA followed by Dunn’s post test was used to compare three or more samples. The Mann–Whitney *U* test was used to compare the differences between two independent groups. The data are presented as means ± standard deviation (SD) or as means ± standard error of mean (SEM). P-values <0.05 were considered significant.

## Results

### Both dipeptides, GK-6 and GK-2, activate TrkA receptors

**Fig. 1:**
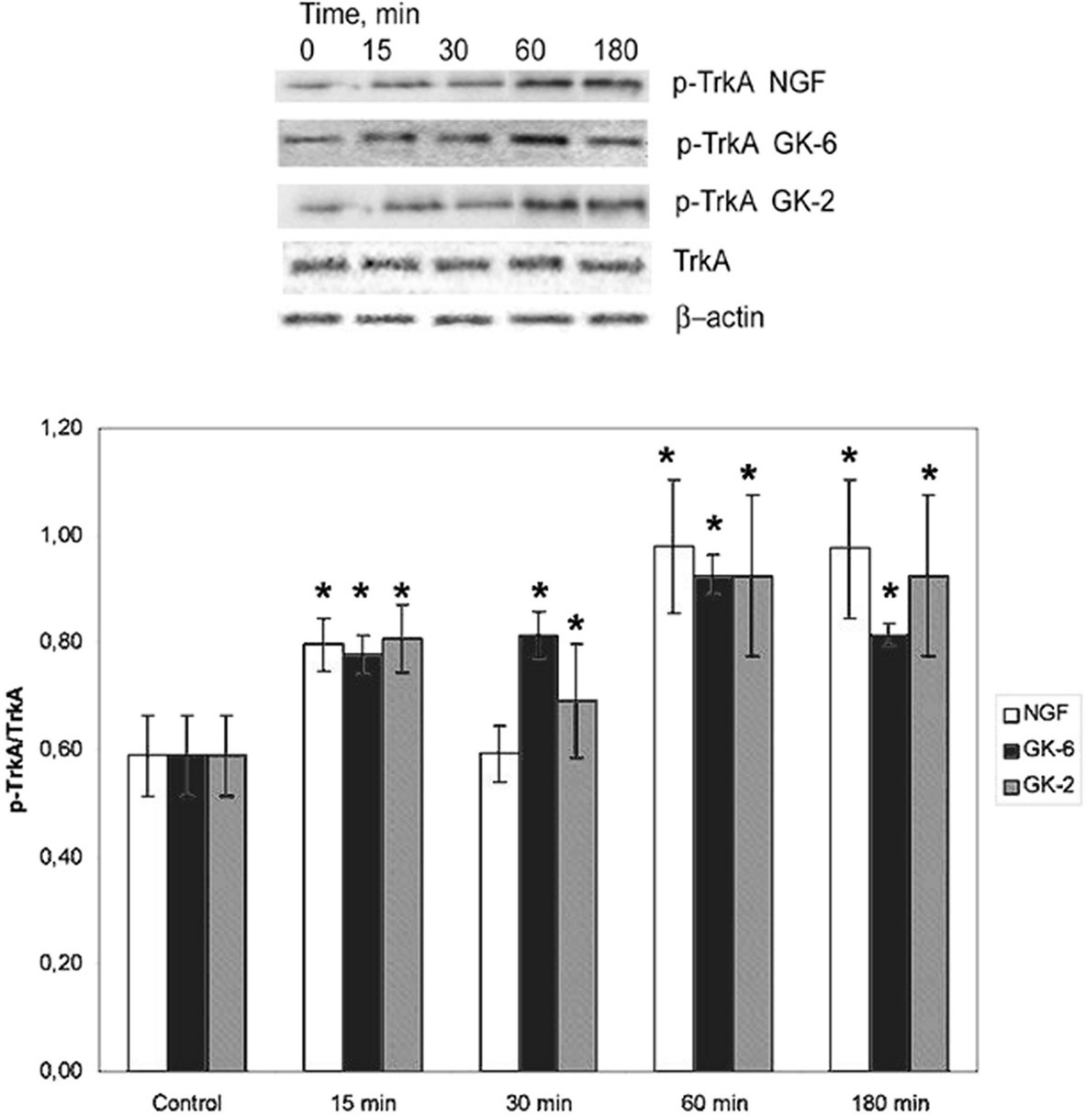
Western blot analysis of TrkA phosphorylation in HT-22 hippocampal neurons at different times (15, 30, 60 and 180 min) after incubation with GK-6 (10^-6^M), GK-2 (10^-8^M) or NGF (10^-9^M). Results were calculated as the ratio of arbitrary densitometric units of phosphorylated target protein to total non-phosphorylated target protein. Total nonphosphorylated target protein arbitrary densitometric units were analyzed separately against β-actin. Data are presented as means±SD of five independent experiments. Differences from the control were significant at *-*р* < 0.05 (Mann–Whitney *U* test)

The dipeptides GK-6 (10^−6^M) and GK-2 (10^−8^M) added to HT-22 hippocampal neurons induced the elevation of TrkA receptor phosphorylation after incubation for 15, 30, 60 and 180 min, similarly to NGF (10^−9^M) (Fig. 1).

### GK-6 and GK-2 exhibit different patterns of PI3K/AKT and MAPK/ERK activation

**Fig. 2:**
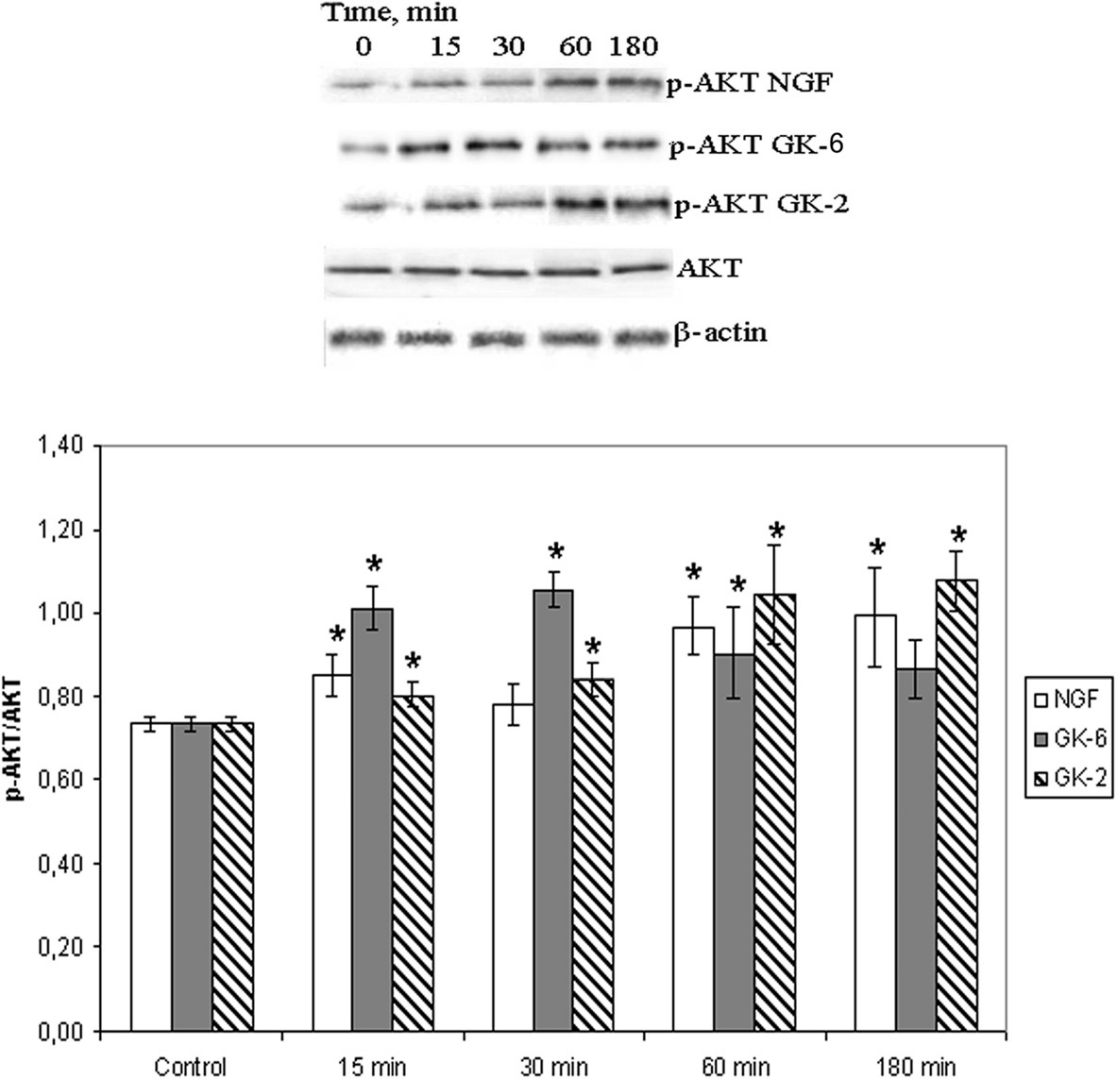
Western blot analysis of AKT 1/2/3 phosphorylation in HT-22 hippocampal neurons at different times (15, 30, 60 and 180 min) after incubation with GK-6 (10^-6^M), GK-2 (10^-8^M) or NGF (10^-9^M). Results were calculated as the ratio of arbitrary densitometric units of phosphorylated target protein to total non-phosphorylated target protein. Total nonphosphorylated target protein arbitrary densitometric units were analyzed separately against β-actin. Data are presented as means±SD of five independent experiments. Differences from the control were significant at *-*р* < 0.05 (Mann–Whitney *U* test)

**Figs. 3:**
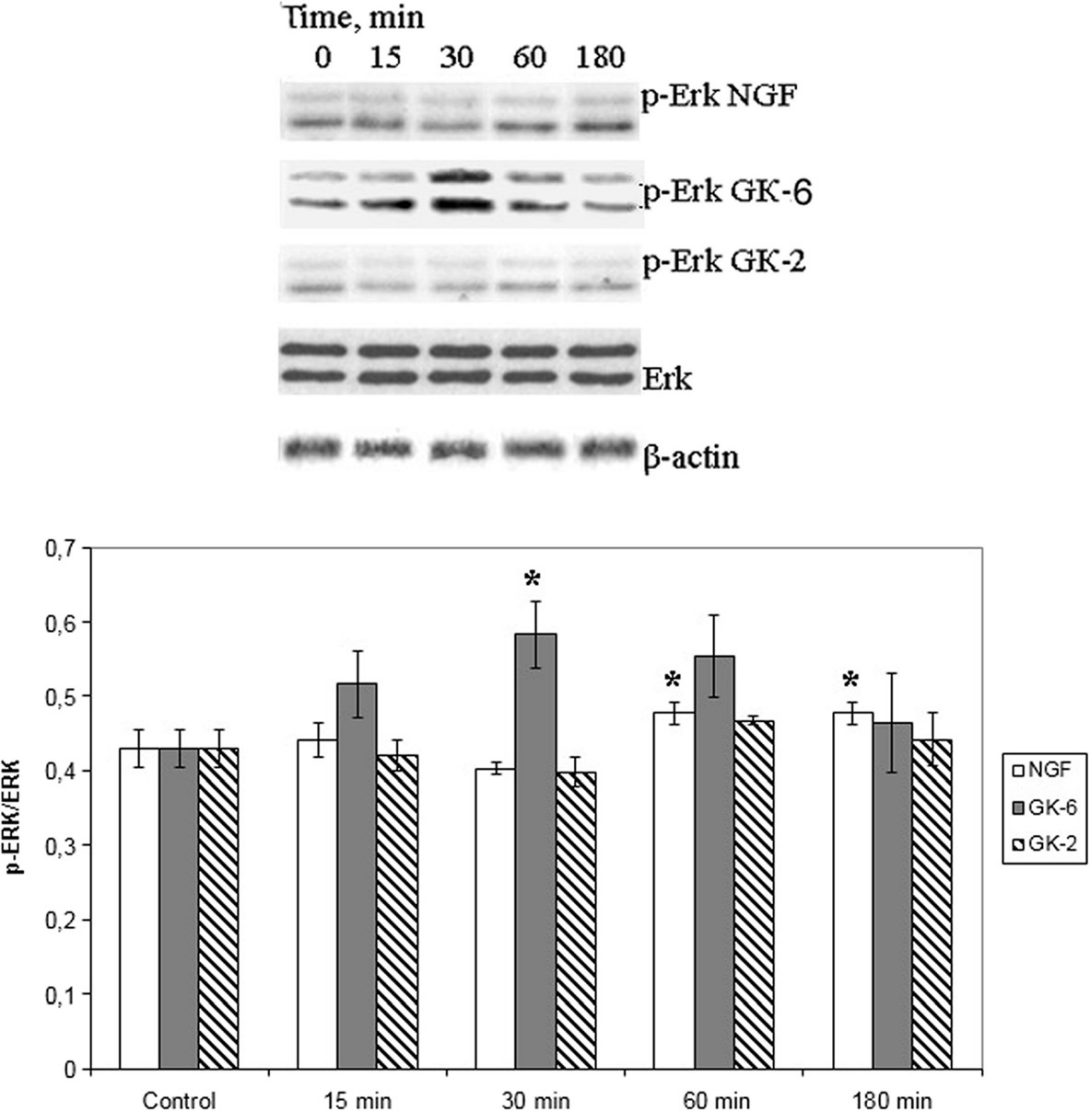
Western blot analysis of ERK 1/2 phosphorylation in HT-22 hippocampal neurons at different times (15, 30, 60 and 180 min) after incubation with GK-6 (10^-6^M), GK-2 (10^-8^M) or NGF (10^-9^M). Results were calculated as the ratio of arbitrary densitometric units of phosphorylated target protein to total non-phosphorylated target protein. Total nonphosphorylated target protein arbitrary densitometric units were analyzed separately against β-actin. Data are presented as means±SD of five independent experiments. Differences from the control were significant at *-*р* < 0.05 (Mann–Whitney *U* test)

ERK activation and AKT activation by GK-6 and GK-2 were assessed using the ERK1/2 and AKT1/2/3 phosphorylation assays. Western blot densitometry analysis revealed that AKT1/2/3 phosphorylation was increased after stimulation of the cells by GK-6 (10^−6^M), GK-2 (10^−8^M) or NGF (10^−9^M) at the time intervals observed for TrkA (i.e., 15, 30, 60, and 180 min incubations) (Fig. 2). GK-6 and NGF induced phosphorylation of ERK1/2. There was no activation of ERK1/2 proteins by GK-2 at any time point (Fig. 3). These data suggest that GK-2 selectively activates PI3K/AKT signaling, whereas GK-6 activates both the PI3K/AKT and MAPK/ERK pathways.

### GK-6 induces the differentiation of PC12 cells

**Fig. 4:**
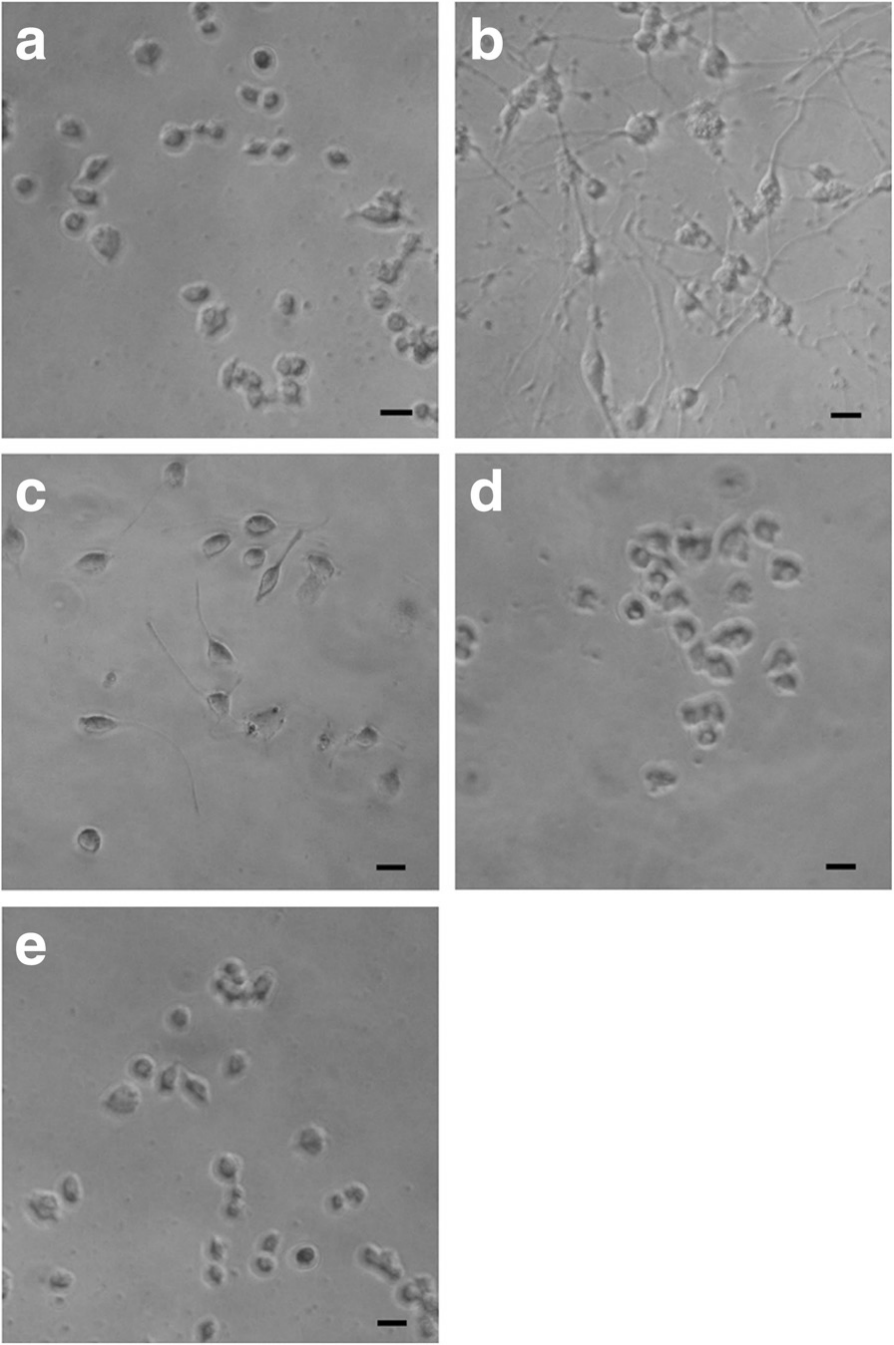
GK-6, similarly to NGF, induces the differentiation of PC12 cells, whereas GK-2 does not. PC12 cells were treated with GK-6 (10^-6^M), GK-2 (10^-5^ and 10^-8^M) or NGF (10^-9^M) 3 times in 48 h. Cell differentiation was studied morphometrically. Magnification: 20×. **a** – untreated control cells, **b** – treatment with NGF, **c** – treatment with GK-6, **d** – treatment with GK-2 (10^-5^ M), **e**-treatment with GK-2 (10^-8^M). The bar in the photomicrographs corresponds to 25 μm

It was established that GK-6 (10^−6^M) and NGF (10^−9^M) induced neurite outgrowth in PC12 cells, whereas GK-2 (10^−5^ and 10^−8^M) had no effect (Fig. 4). These findings are consistent with previous studies demonstrating that GK-6 activates the MAPK/ERK signaling pathway involved in cell differentiation, whereas GK-2 selectively activates the PI3K/AKT pathway.

### LY294002, a selective inhibitor of PI3K, fully abolishes the neuroprotective activity of GK-2

**Fig. 5:**
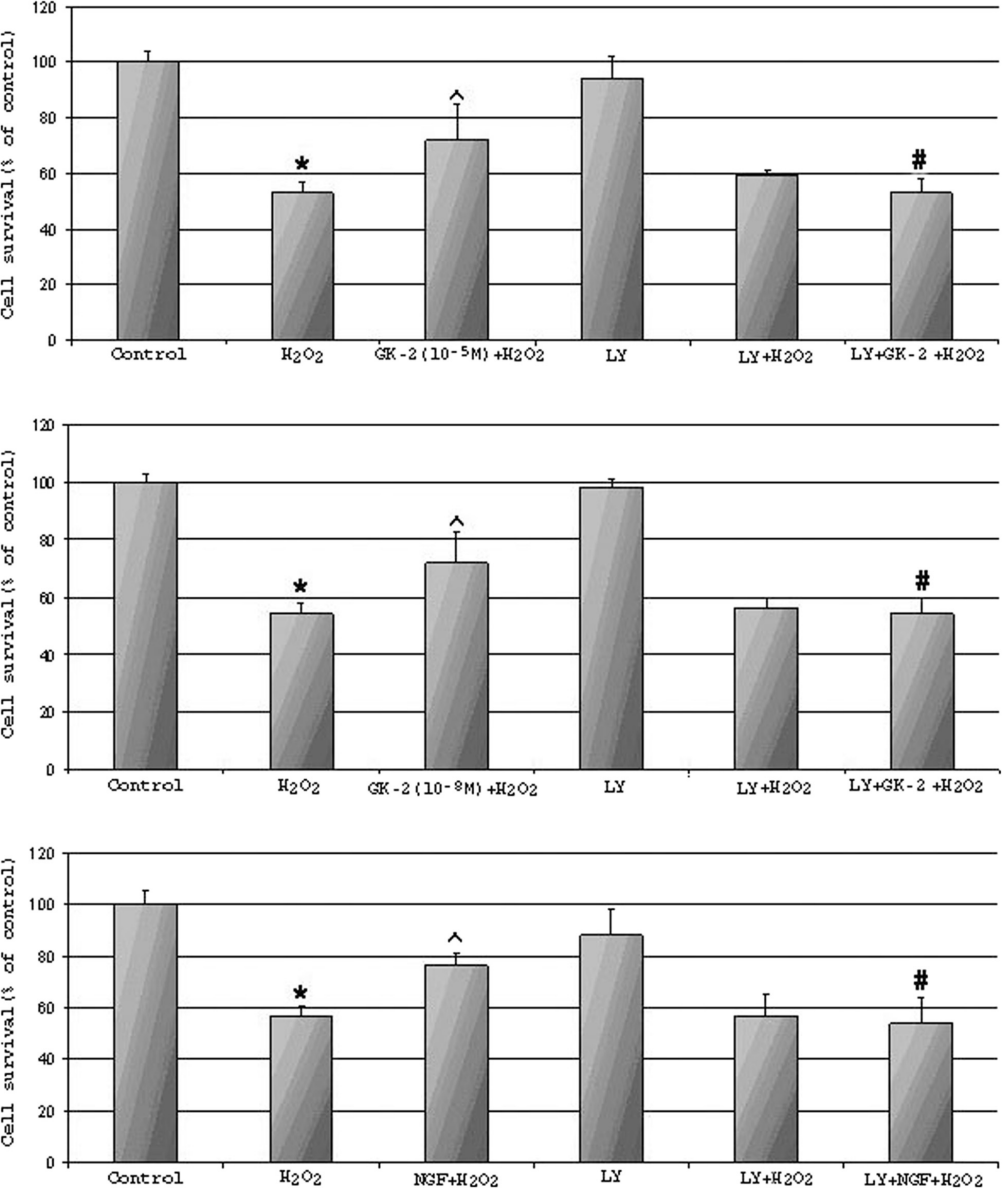
Effects of the AKT kinase inhibitor LY294002 on the neuroprotection provided by GK-2 or NGF against H_2_O_2_-induced oxidative stress in HT-22 cells. Hippocampal cells, HT-22, were pre-incubated with LY294002 (100 μM) for 30 min before treatment with GK-2 (10^-5^M and 10^-8^M) or NGF (10^-9^M). Cell survival after a 30-min exposure to H_2_O_2_ was measured by assessment of MTT metabolism. Data are presented as means±SD. * *p* < 0.05 compared with control, ^ *p* < 0.05 compared with H_2_O_2_ group, # *p* < 0.05 compared with corresponding H_2_O_2_ + GK-2 or NGF + GK-2 (Kruskal–Wallis ANOVA test with Dunn’s post hoc)

**Fig. 6:**
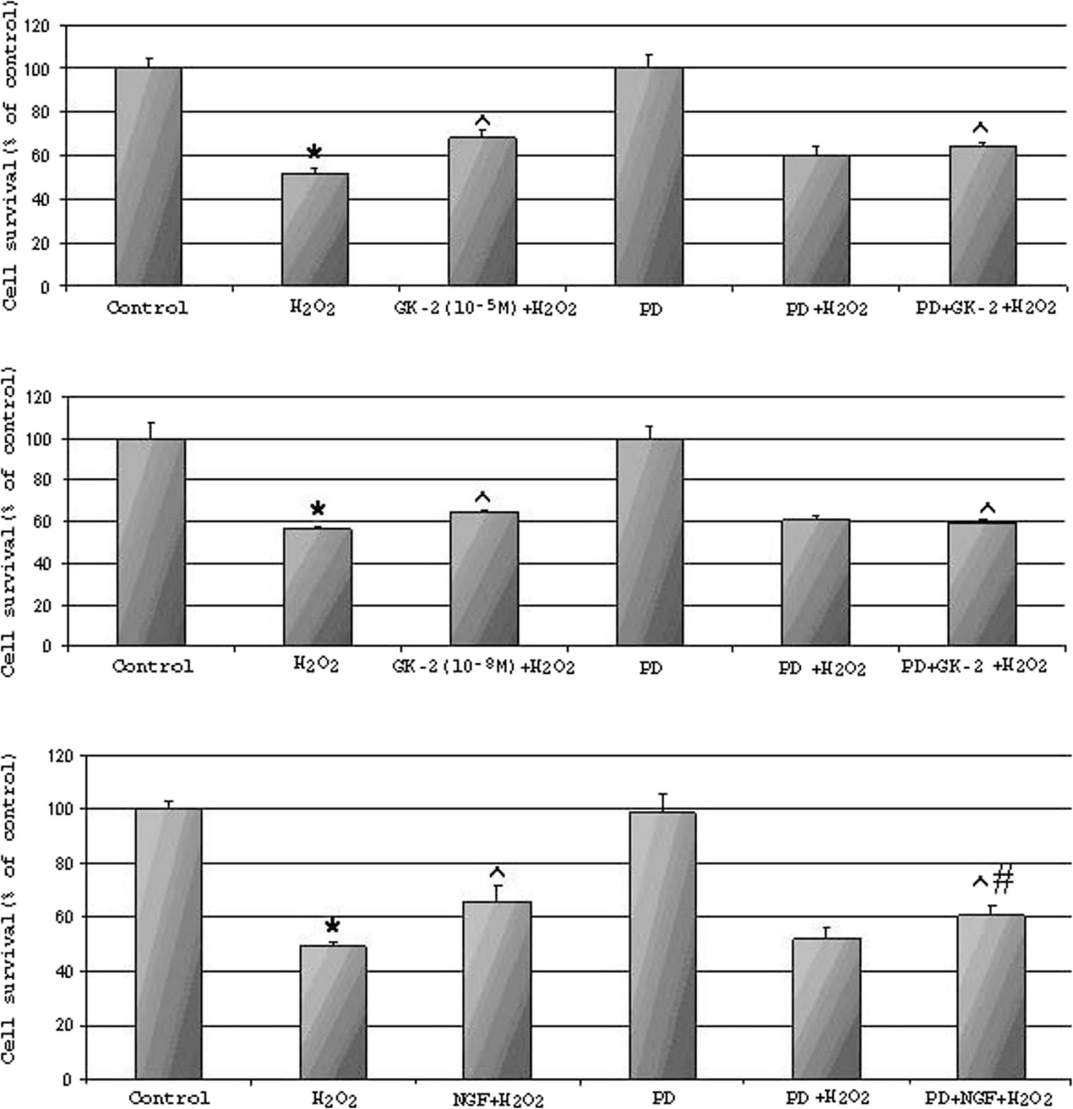
Effects of the MAPK kinase inhibitor PD98059 on the neuroprotection provided by GK-2 or NGF against H_2_O_2_-induced oxidative stress in HT-22 cells. Hippocampal cells, HT-22, were pre-incubated with LY294002 (100 μM) for 30 min before treatment with GK-2(10^-5^M and 10^-8^M) or NGF (10^-9^M). Cell survival after a 30-min exposure to H_2_O_2_ was measured by assessment of MTT metabolism. Data are presented as means±SD. * *p* < 0.05 compared with control, ^ *p* < 0.05 compared with H_2_O_2_ group, # *p* < 0.05 compared with corresponding H_2_O_2_ + GK-2 or NGF + GK-2 (Kruskal–Wallis ANOVA test with Dunn’s post hoc)

To confirm the involvement of the PI3K/AKT pathway in the neuroprotective effects of GK-2, we pretreated HT22 cells with LY294002 (a selective inhibitor of PI3K) or PD98059 (a specific inhibitor of MAPK) followed by GK-2 or NGF and then H_2_O_2_. As shown in Fig. 5, compound LY294002 fully blocked the neuroprotective effects of both NGF and GK-2. Under the same conditions, PD98059 had practically no effect on the development of the neuroprotective action of GK-2 and partly abolished the neuroprotective effect of NGF (Fig. 6). These results indicate that GK-2 enhances cell survival through the increased activation of the PI3K/AKT pathway. Interestingly, LY294002 almost fully prevented the neuroprotective action of NGF, whereas PD98059 only partially prevented the neuroprotection. These results can be explained by the data indicating that NGF-mediated survival is associated mainly with the PI3K/AKT pathway and, to a much lower extent, with MAPK/ERK signaling [2].

### Different effects of GK-6 and GK-2 on pain sensitivity in rats

**Fig. 7:**
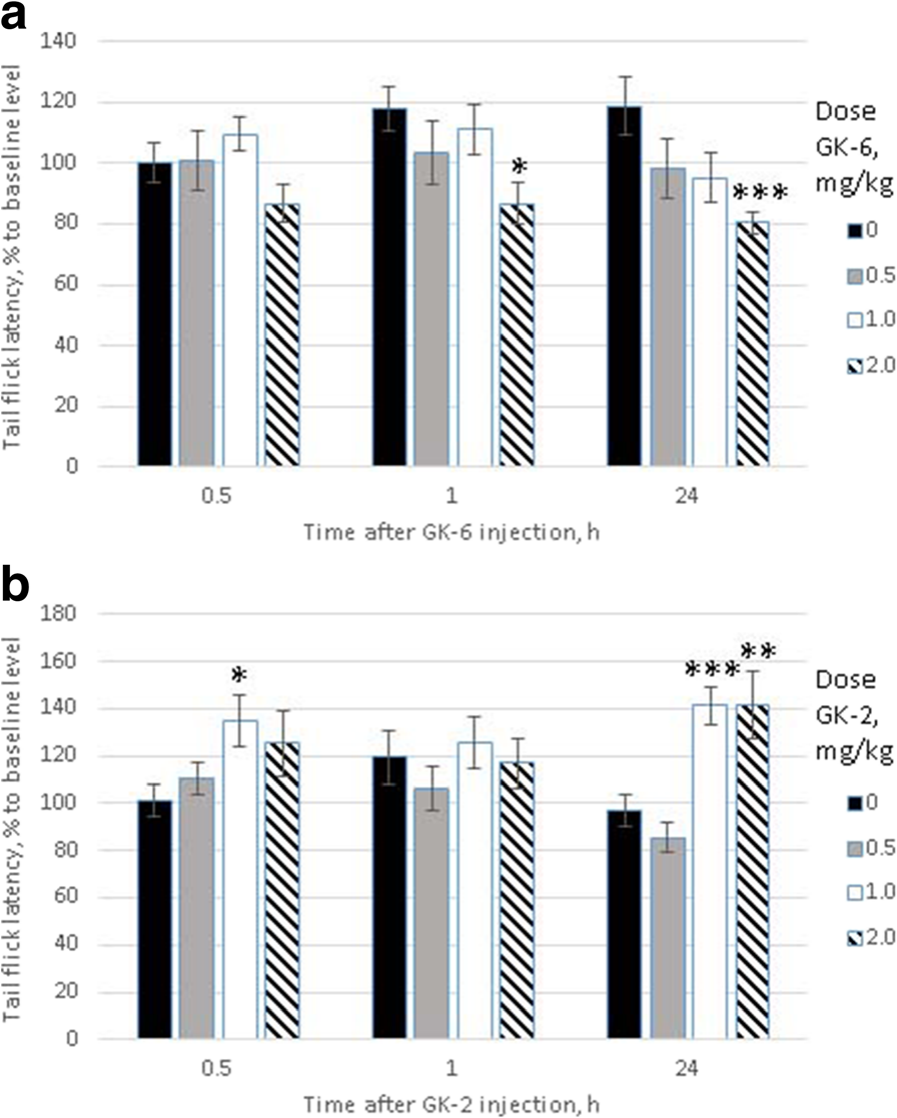
Effect of GK-6 (**a**) and GK-2 (**b**) on the pain thresholds in rats measured by the immersion “tail flick” test. The dipeptide GK-2 was administered i.p. at doses of 0.5, 1 and 2 mg/kg. Noxious stimuli were applied by immersing the distal third of each rat's tail in hot water (55±0.2ºC). Tail flick latency was measured before (baseline level) and 30, 60 min and 24 h after GK-2 administration. The time intervals were selected based on data related to NGF-induced hyperalgesia. Data are presented as means±S.E.M. * *p* < 0.05, ** *p* < 0.01, *** p .0.001 compared to the control group (Mann–Whitney *U* test)

The dipeptide GK-6 at a dose of 2.0 mg/kg significantly decreased the pain threshold at 1 h (by 30.0 %) and 24 h (by 38.0 %) after injection compared to the control group (Fig. 7a). As shown in Fig. 7b, GK-2 at all doses tested did not have any hyperalgesic effect in the tail flick test. In fact, this peptide had the opposite effect: 30 min and 24 h after administration at a dose of 1.0 mg/kg, it significantly increased the pain threshold relative to the control group by 34.0 % and 44.0 %, respectively; and at a dose of 2.0 mg/kg, it increased the pain threshold by 44.0 % relative to the control group 24 h after administration. The development of an analgesic effect at the same time intervals as the hyperalgesic effect of NGF [17] suggests that GK-2 may be a functional agonist/antagonist of the neurotrophin.

### GK-6 and GK-2 have no influence on the body weight of rats

**Fig. 8:**
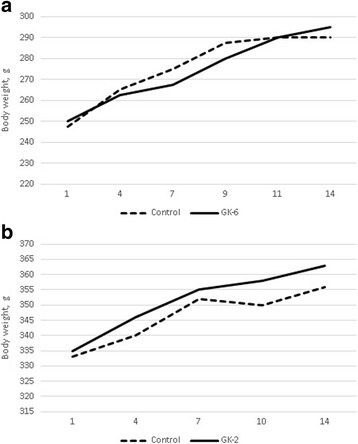
Chronic administration of GK-6 (**a**) and GK-2 (**b**) had no influence on the rats’ body weight. GK-6 (2.0 mg/kg, i.p.) or GK-2 (0.5 mg/kg, i.p.) were injected daily for 14 days. Control animals received injections of distilled water. The body weight of the rats was measured every three-four days during the experiment. The horizontal axis shows the days of the experiment, the vertical axis shows the average body weight of animals for each group. Data are presented as means±SEM

Preclinical and clinical trials have shown that NGF treatment is accompanied by body weight loss [18]. Unlike NGF, daily administration of GK-6 or GK-2 at the most effective doses (2.0 and 0.5 mg/kg/day, respectively, i.p.) for 2 weeks did not affect the body weight of the rats (Fig. 8).

## Discussion

Successful attempts to modulate TrkA signaling patterns by specific ligands have been previously described in the literature. Saragovi H.U. et al. [19] reported that NGF in association with anti-NGF antibodies directed against the C termini of NGF promoted survival but not neurite outgrowth. Mechanism-selective recombinant NGF muteins that did not support neuritogenesis but maintained the survival response were designed and characterized by Maharapta S. et al. [20]. Capsoni S. et al. [21] developed a “painless” NGF variant by making a point mutation at residue R100, inspired by the human genetic disease HSAN V (Hereditary Sensory Autonomic Neuropathy Type V).

We found that dimeric dipeptide mimetics designed based on the NGF loop 1 and 4 β-turn sequences have different patterns of signal transduction and different profiles of biological activity. It was revealed by Western blot assays that the dipeptide mimetic of NGF loop 1 (GK-6) increased the levels of AKT and ERK phosphorylation, whereas the dipeptide mimetic of NGF loop 4 (GK-2) only increased the level of AKT phosphorylation. These data suggest that GK-6 activates the PI3K/AKT and MAPK/ERK pathways, whereas GK-2 selectively activates the PI3K/AKT pathway. Of the two dipeptides, only GK-6 exhibits differentiating activity in PC12 cells. It is known that cell differentiation through the TRKA receptor is associated with MAPK/ERK signaling [2]. Thus, the result that GK-2 does not induce differentiation of PC12 cells agrees with the result that the dipeptide does not activate the MAPK/ERK pathway. In addition, we established that the neuroprotective activity of GK-2 was fully abolished by a selective inhibitor of PI3K (LY294002) but not a MAPK kinase inhibitor (PD98059). These results indicate that GK-2 selectively activates the PI3K/AKT pathway.

The main side effects of NGF are pain and weight loss [1]. It was shown, using the tail flick test in rats, that GK-6 induced a significant decrease in the pain threshold 1 and 24 h after injection. These time points coincide with the peaks of NGF-induced hyperalgesia [17]. By contrast, GK-2 did not show any hyperalgesic effect. This result suggests that the MAPK/ERK pathway is involved in the development of hyperalgesia mediated by the TrkA neurotrophin receptor, whereas the selective activation of the PI3K/AKT pathway does not cause an increase in pain sensitivity. Moreover, in our experiments, GK-2 had the opposite effect: 30 min and 24 h after administration, it increased the pain threshold relative to both the control group and the baseline level. Importantly, when administered chronically, neither dipeptide caused weight loss in rats. For instance, daily i.p. administration of NGF resulted in significant weight loss in rats, approximately 15 % compared with control animals at the 6 day and almost 20 % at the 30 day of treatment [18]. The fact that GK-6 and GK-2 have no effect on body weight means that the activation of pathways other than the PI3K/AKT and MAPK/ERK pathways is required to produce effects on body weight.

## Conclusions

The current results suggest that the most exposed outside fragment of the NGF 4th loop β-turn is the structural determinant of selective AKT-kinase pathway activation. Furthermore, our data support the hypothesis that the selective activation of the AKT-kinase pathway is not associated with hyperalgesia, one of the main side effects of NGF, whereas the activation of both the AKT and MAP-kinase pathways generates nociceptive effects.

The advantageous pharmacological properties of the dipeptide GK-2 make it a promising NGF-like neuroprotective therapeutic agent free of the main side effects of the neurotrophin.

## Abbreviations

ERK: Extracellular signal-regulated kinase
MAPK: MEK1, MEK2, mitogen-activated protein kinases
NGF: Nerve growth factor
PI3K: Phosphatidylinositol 3-kinase
TrkA: Tyrosine kinase A
